# Analgesic efficacy of intraperitoneal local anaesthetic instillation (IPLA) in laparoscopic bariatric surgery: a systematic review and meta-analysis

**DOI:** 10.1186/s44158-026-00345-3

**Published:** 2026-01-26

**Authors:** Maria Luisa Garo, Sabrina Migliorelli, Flavia Comitini, Massimiliano Ricci, Alessandro Strumia, Alessandro Ruggiero, Marta Di Folco, Fabio Costa, Lorenzo Schiavoni, Alessia Mattei, Fedra Lavorante, Rita Cataldo, Massimiliano Carassiti, Felice Eugenio Agrò, Giuseppe Pascarella

**Affiliations:** 1 Anaesthesia and Intensive Care Research Unit, Università Campus Bio-Medico, Via Álvaro del Portillo 21, Rome, 00128 Italy; 2 Anaesthesia and Intensive Care Operative Unit, Fondazione Policlinico Universitario Campus Bio-Medico, Via Álvaro del Portillo 200, Rome, 00128 Italy

**Keywords:** Intraperitoneal local anaesthetic, Bariatric surgery, Postoperative pain, Opioid consumption, Laparoscopic surgery, Multimodal analgesia

## Abstract

**Background:**

Laparoscopic bariatric surgery is effective for weight loss but often requires opioids for postoperative pain management, possibly increasing complications. Intraperitoneal local anaesthetic (IPLA) instillation may help to reduce pain and opioid use, though its efficacy remains unclear. This systematic review and meta-analysis aims to evaluate the impact of IPLA on postoperative pain management and opioid consumption in patients undergoing laparoscopic bariatric surgery.

**Methods:**

Following PRISMA guidelines, a systematic search of PubMed, Scopus, Web of Science and Cochrane Library (up to July 31, 2024) identified randomized controlled trials (RCTs) comparing IPLA with placebo or other analgesics. Primary outcomes were postoperative pain scores; secondary outcomes included opioid consumption, hospital length of stay (LOS) and incidence of postoperative nausea and vomiting (PONV). Risk of bias was assessed using Cochrane RoB2, and a random-effects model was used for statistical analysis.

**Results:**

Eight RCTs (*n* = 875) showed IPLA significantly reduced pain in the first 4 h (SMD: − 1.46, 95% CI: − 2.08 to − 0.85, *p* < 0.001) and 4–8 h postoperatively (SMD: − 1.16, 95% CI: − 1.94 to − 0.37, *p* < 0.001), with no effect beyond 8 h. IPLA reduced additional analgesic use (RR: 0.41, 95% CI: 0.25–0.66, *p* < 0.001) but without significant impact on LOS or PONV. Due to heterogeneity in opioid consumption reporting, a pooled analysis was not feasible.

**Conclusion:**

IPLA effectively reduces early postoperative pain and opioid demand in laparoscopic bariatric surgery, though long-term benefits remain uncertain. Further high-quality RCTs are needed to establish optimal administration techniques and assess their broader clinical benefits.

**Supplementary Information:**

The online version contains supplementary material available at 10.1186/s44158-026-00345-3.

## Introduction

Obesity is a major global public health challenge, closely associated with increased morbidity and mortality [1, 2]. Adult obesity rates more than doubled worldwide between 1990 and 2022 with prevalence increasing twofold in women (from 8.8 to 18.5%) and nearly tripling in men (from 4.8 to 14%) [3]. If these trends continue, it is projected that by 2030, 51% of the adult population will be classified as obese [4].

In this context, bariatric surgery plays a critical role in facilitating substantial and sustained weight loss [5–7]. The overall volume of bariatric surgery has increased by 60% from 2011 to 2018, with sleeve gastrectomy (SG) demonstrating a notable 451% increase [8]. Over the years, bariatric surgery has evolved from open procedures to laparoscopic techniques, offering improved outcomes through minimally invasive approaches [9–11]. A worldwide survey on bariatric surgery published in 2015 revealed that 468,609 bariatric procedures were performed worldwide in 2013, 95.7% of which were already carried out with a laparoscopic approach [12]. Despite the benefits of minimally invasive techniques, patients with obesity are at greater risk of poorly controlled postoperative pain compared with non-obese individuals [13, 14], often resulting in moderate to severe postoperative pain, which is an independent predictor of prolonged hospital stay and delayed postoperative recovery [15, 16].

Moreover, a high opioid demand is strictly related to post surgery complications such as sedation, postoperative nausea and vomiting (PONV), gastrointestinal paralysis and respiratory depression/hypoventilation. In particular, postoperative respiratory complications may occur more frequently in individuals with obesity, among whom the prevalence of obstructive sleep apnoea (OSA) is estimated to be as high as 45% [17].

Furthermore, an inadequately managed pain significantly increases the risk of developing chronic pain [18], and of long-term opioid use [19, 20]. Multimodal opioid-sparing analgesic strategies, including regional anaesthesia (RA) techniques, have proven effective in controlling pain while minimising opioid consumption and its associated side effects, such as postoperative sedation, nausea, gastrointestinal paralysis and respiratory suppression [21–23]. This approach is consistent with Enhanced Recovery After Surgery (ERAS) protocols [24, 25], which are specifically tailored to bariatric surgery (ERABS) [26, 27] and aim to optimize recovery and minimise postoperative opioid administration.

Various RA techniques have proven effective in managing postoperative pain in patients undergoing laparoscopic abdominal surgery, including those undergoing bariatric surgery [28–30]. For instance, the epidural anaesthesia [31, 32], although effective, can be associated with severe side effects, such as hypotension, post-dural puncture headache and spinal hematoma with neurological symptoms. Other peripheral RA techniques, such as fascial blocks (transversus abdominis plane—TAP—block [33, 34], quadratus lomborum block—QLB [35] and erector spinae plane—ESP—block [36]) and port-site infiltration (PSI) [37] have been shown to be effective in managing somatic pain but not visceral pain originating from the peritoneal innervation. Moreover, fascial blocks can be technically challenging in patients with a high body mass index (BMI), even when using ultrasound (US) guidance [38, 39].

Intraperitoneal local anaesthetic (IPLA) instillation consists of the administration of local anaesthetics into the intraperitoneal cavity and has already been shown to be an effective strategy to reduce pain and opioid consumption after laparoscopic surgery [23, 40, 41]. This benefit has been observed in both general [42–44] and gynaecologic procedures [45], suggesting a common pain mechanism.

Recently, studies have been conducted on the efficacy of IPLA in laparoscopic bariatric surgery, but the evidence remains inconclusive [46–55]. For this reason, the aim of this systematic review and meta-analysis is to examine the impact of IPLA on postoperative analgesia and opioid consumption in patients undergoing laparoscopic bariatric surgery.

## Methods

This systematic review was conducted according to the Preferred Reporting Items for Systematic Reviews and Meta-Analyses (PRISMA) guidelines [56]. The protocol is registered on the PROSPERO database (ID: CRD42024564878; Date of submission: July 22, 2024).

The components of PICO were (Population) adult patients undergoing laparoscopic bariatric surgery; (Intervention) IPLA instillation; (Comparator) other anaesthetic techniques or placebo; and (Outcome) postoperative pain, hospital length of stay (LOS), cumulative opioid consumption in the first 24 h after surgery and incidence of PONV.

### Literature search

A systematic literature search of Scopus, PubMed–Medline, Web of Science and Cochrane Library was performed until 31 st July 2024, using the following search strategy ((((((“intraperitoneal”) OR “intra peritoneal”)) AND ((((“instillation”) OR “infiltration”) OR “injection”) OR “administration”)) AND ((((((((((“local anesthetic”) OR “local anaesthetic”) OR “local anesthesia”) OR “local anaesthesia”) OR “lidocaine”) OR “lignocaine”) OR “mepivacaine”) OR “ropivacaine”) OR “bupivacaine”) OR “levobupivacaine”)) AND (((((“bariatric surgery”) OR “sleeve gastrectomy”) OR “gastric bypass”) OR “roux en y”) OR “gastric banding”)) AND ((“laparoscopic”) OR “laparoscopy”). For further details, see Supplementary Materials.

### Eligibility criteria

Peer-reviewed randomised control trials (RCTs) or abstracts or RCT written in English investigating the effectiveness of IPLA instillation (IPLA group—IPLA) performed by the surgeon were considered compared to patients who did not receive IPLA or received a placebo (control group—CG). Studies considering the exclusive use of general anaesthesia (GA) or in whom IPLA was combined with other analgesic techniques, studies investigating intraperitoneal administration of medications not classified as local anaesthetics, but that have local anaesthetic properties at high doses (e.g. ondansetron, ketamine) were excluded. We also excluded trial protocols, studies including paediatric patients (< 18 years old) or urgent/emergent surgeries, conference abstracts (i.e. where the full text could not be sourced), letters to the editor and short communications.

### Study selection

**Fig. 1:**
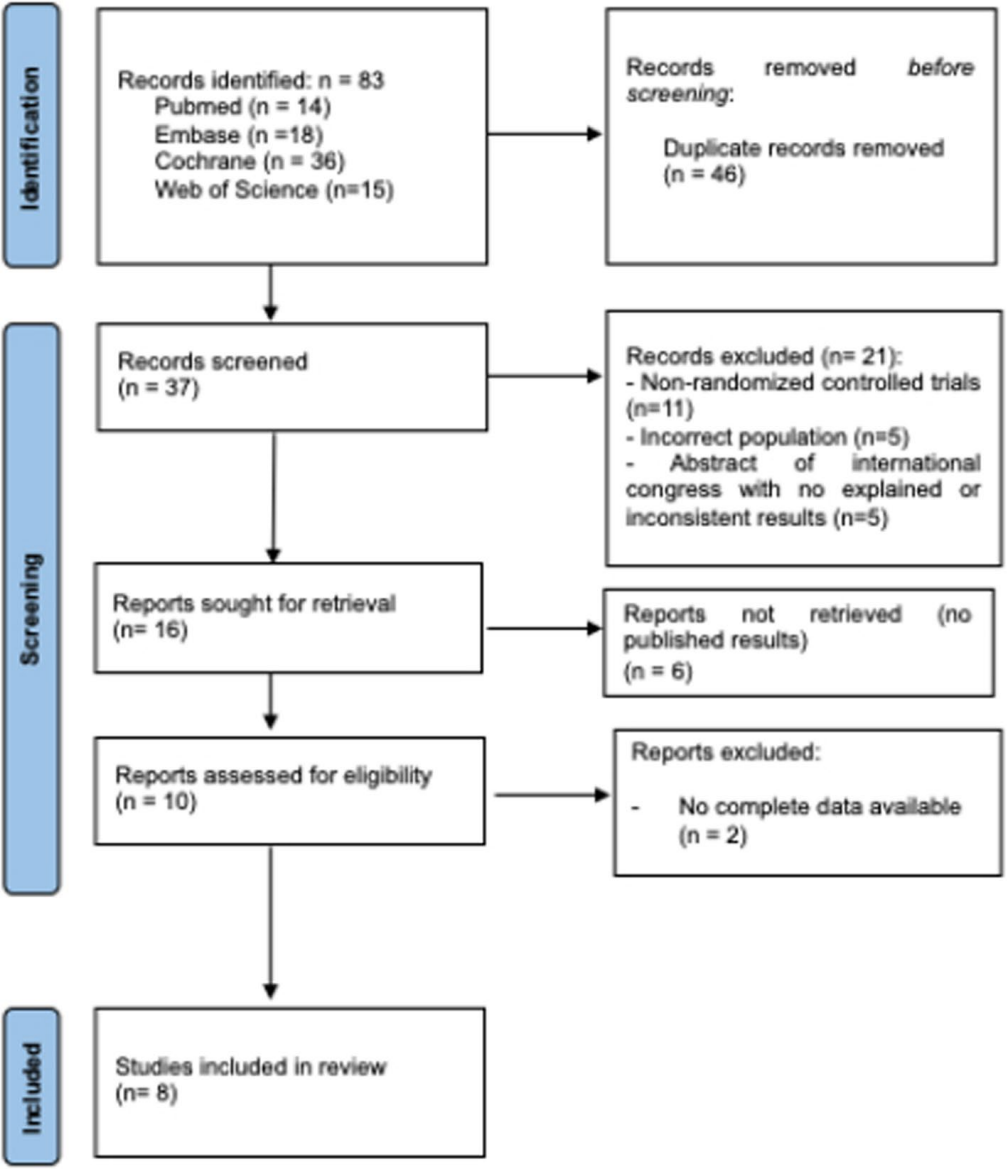
PRISMA flowchart. This flowchart illustrates the study selection process according to PRISMA (Preferred Reporting Items for Systematic Reviews and Meta-Analyses) guidelines

The study selection was conducted using Rayyan software [57]. After removing duplicates, an initial screening of the title and the abstract of eligible studies was performed by three reviewers (S.M., F.C and M.R.). In case of disagreements, the consensus of a fourth reviewer (G.P.) was asked. Finally, two reviewers (S.M. and G.P.) read the full articles to assess the studies for inclusion in this review. The number of articles excluded and included were recorded and reported in a PRISMA flowchart (Fig. 1).

### Screening process and data extraction

Three independent reviewers (S.M, F.C. and M.R.) conducted data extraction. Data regarding study characteristics [first author, publication year, study period, country, study design, kind of study (monocentric or multicentric), level of evidence (LOE)], surgery characteristics (type, indications, acuity, duration and if revision surgery), patient demographic characteristics [sample size in total and in intervention and control groups, age, gender, BMI, and the American Society of Anesthesiologists (ASA) physical status classification], history of chronic pain conditions, pre-operative pain medication use, pre-operative chronic opioid and non-opioid use and characteristics of IPLA delivery (type, timing, dose, method and site of administration) were extracted.

When continuous data were reported as the median and range (or inter-quartile range), estimates of the mean and standard deviation (SD) were derived using validated methods [58, 59]. The accuracy of the extracted data was independently validated by a second author (M.L.G.), with any discrepancies resolved through discussion.

### Outcome measures

The primary outcomes of our study were overall pain scores (in the 0–4 h, 4–8 h, 8–12 h, over 12 h postoperatively). Pain scores were assessed using various scales, including the Visual Analogue Scale (VAS) and Numeric Rating Scale (NRS). When pain scores were reported at multiple time points within a given interval, the highest value within that interval was used for analysis (e.g. if pain was reported at 1-, 2- and 4-h post-surgery, and the 4-h pain score was the highest, it was used for the 0–4 h pain outcome analysis). Secondary outcomes included LOS, cumulative opioid consumption in the first 24 h after surgery, incidence of PONV and postoperative complication rate [60]. Opioid consumption was measured in intravenous (i.v.) morphine equivalent doses (MED) [61].

### Risk of bias

The quality of the included studies was assessed independently by three independent reviewers (M.L.G., S.M. and G.P.) using the Revised Cochrane Risk-of-bias tool for randomised trials (RoB2) [62]. The tool assesses bias in five specific domains: randomisation process, deviations from intended interventions, missing outcome data, measurement of the outcome and selection of the reported result. The overall risk of bias for each study was determined according to the RoB2 guidelines. Potential disagreements were resolved by discussion and consensus among all authors. The “Robvis” tool was utilized to generate the traffic light plot and the risk of bias summary plot in accordance with Cochrane recommendations [63].

### Statistical analysis

All data from the eligible articles were summarized and used for the meta-analysis. A random effects model using the Sidik–Jonkman estimator was applied to calculate the pooled effect size for each outcome. This model was selected a priori based on the expected heterogeneity between studies due to differences in surgical techniques, patient characteristics and inclusion and exclusion criteria. For continuous outcomes such as postoperative pain and LOS, we used the standardised mean difference (SMD). This choice was necessary because pain was measured with different scales (e.g. VAS 0–10 vs. 0–100) and the SMD allows standardisation across heterogeneous measurement instruments. For binary outcomes (e.g. use of analgesia on demand, PONV), we calculated risk ratios (RR) with 95% confidence intervals. Due to substantial heterogeneity in the reporting of opioid consumption (e.g. different medications, dosages, routes of administration, and time periods), a pooled estimate for this outcome could not be determined and results were reported descriptively. Heterogeneity was assessed using the Cochrane *Q* test and the I^2^ statistic following the Cochrane Handbook for Systematic Reviews of Interventions (Chapter 10, Section 10.9). Potential sources of heterogeneity, such as outliers, were examined using Galbraith plots or L’Abbé plots, depending on the type of data. Cumulative meta-analyses and leave-one-out sensitivity analyses were performed to assess the robustness of the results. Publication bias was not assessed as the number of studies included in each analysis did not reach the minimum threshold of ten. All analyses were performed using STATA 18 (StataCorp, College Station, TX, USA).

### Level of certainty

To assess overall confidence in the estimates of effect for each outcome, we used the GRADE (Grading of Recommendations Assessment, Development and Evaluation) method. Evidence was synthesized using GRADEpro GDT software and graded according to five domains: Risk of bias, inconsistency, indirectness, imprecision and other considerations.

## Results

Eighty-three studies were retrieved by databases and registers (PubMed: *n* = 14, EMBASE: *n* = 18, Cochrane Library: *n* = 36, Web of Science: *n* = 15). After removing duplicates, thirty-seven studies were screened by title and abstract, of which only 10 were eligible for full-text analysis. Eight of these were included in the study, comprising a total of 875 patients (IPLA: 435, CG: 440).

### Studies’ characteristics

The eight included studies, published between 2005 and 2023, were conducted in Iran [46, 52], Canada [48], Australia [49], Bahrain [50], Spain [51], USA [54] and Malaysia [55]. The average age of the participants ranged from 28.9 years [46] to 46.5 years [51]. Female participants were predominantly represented: in all included studies, the proportion of women was at least over 67%. Mean BMI was 44.9 and only two studies reported that the patients had a pain syndrome [48, 49]. The duration of surgery ranged from 62 min [50] to more than 2 h [48]. Pain—the primary outcome of all included studies—was measured at different times and at different intervals. In two studies, pain was measured up to 48 h after surgery [48, 49] and in the oldest study [54], pain was assessed at 1 h postoperatively and then at 4-h intervals until the patient was discharged. Full information on the studies’ characteristics and surgical details can be found in Tables 1 and 2.

**Table 1:** Studies’ characteristics

Study (year)	Country	Study design	Monocentric	LOE	Observation period	Sample size	Mean age (years)	Gender (M/F)	Timepoints	Secondary outcomes
Alamdari et al (2018) [46]	Iran	RCT	Yes	I	Jun 2015-Sept 2016	IPLA: 60 CG: 60	IPLA: 29.8 ± 5.7 CG: 28.9 ± 6.6	IPLA:M 16 (26.7%)—F 44 (73.3%) CG: M 18 (30%)—F 42 (70%)	• 6 h • 12 h • 24 h	• PONV • PO oral intake • LOS
Jarrar et al (2021) [48]	Canada	RCT	Yes	I	Jul 2014—Feb 2025	IPLA: 46 CG: 46	IPLA: 44.4 ± 9.42 CG: 45.1 ± 9.44	IPLA: M 8 (17)—F 38 (83%) CG: M 7 (15%)—F 39 (85%)	• 1–8 h • 9–24 h • 25–48 h	• Opioid analgesic use • PEF • 6MWT
Kaur et al (2022) [49]	Australia	RCT	Yes	I	Nov 2018 -Nov 2020	IPLA: 50 CG: 54	IPLA: 44 (IQR 30.3–49.8) CG: 34 (IQR 28–49)	IPLA: M 10 (20%)—F 40 (80%) CG: M 12(22.2%)—F 42 (77.8%)	• PACU • 1 h • 2 h • 4 h • 6 h • 24 h • 48 h	• Postoperative analgesia and antiemetic use • LOS • Reoperation • Readmission • Complications • Mortality
Omar et al (2019) [50]	Bahrain	RCT	Yes	I	Jul 2018-Dec 2018	IPLA: 50 CG: 50	IPLA: 34.14 ± 13 CG: 34.14 ± 13	IPLA: M 16 (32%)—F 34 (68%) CG: M 17 (34%)—F 33 (66%)	• PACU • 2 h • 4 h • 6 h • 12 h • 24 h	• Opioid use • PONV, • Rescue analgesia • Shoulder tip pain
Ruiz-Tovar et al (2016) [51]	Spain	RCT	Yes	I	Jan 2015 -Nov 2015	IPLA: 55 CG:55	IPLA: 44.6 ± 10.6 CG: 46.5 ± 9.8	IPLA: M 17 (31%)—F 38 (69%) CG: M 18 (32%)—F 37 (68%)	24 h	• Morphine needs • PONV • Early taking of fluids by mouth • Early mobilization ability • PO complications • Mortality • Length of hospitalization • Acute phase reactants 24 h after surgery
Safari et al (2019) [52]	Iran	RCT	Yes	I	NR	IPLA: 54 CG: 52	IPLA: 37.5 ± 9.2 CG: 36.4 ± 11.3	IPLA: M 11 (20.4%)—F 43 (76.6%) CG: M 9 (17.3%)—F 42 (82.7%)	• 1 h • 4 h • 8 h • 24 h	• Opioids • Use PO (pethidine and morphine)
Symons et al (2007) [54]	USA	RCT	Yes	I	Oct 2004- Mar 2005	IPLA: 65 CG: 68	IPLA: 44.3 ± 1.4 CG: 44.1 ± 1.5	IPLA: M 8 (12.3%)—F 55 (87.7%) CG: M 16 (23.5%)—F 51 (76.5%)	1 h and every 4 h until discharge	• Narcotic use • Incentive spirometer volumes • Antiemetics use • LOS
Zheng et al (2023) [55]	Malaysia	RCT	Yes	I	Nov 2020-May 2021	IPLA: 55 CG: 55	IPLA: 46 (IQR 9) CG: 40 (IQR 13)	IPLA: M 15 (27%)—F 40 (73%) CG: M 16 (29%)—F 39 (71%)	• 2 h • 4 h • 6 h • 12 h • 24 h	• Rescue analgesic • PONV • PO respiratory efforts

*6MWT* six minute walk test, *CG* control group, *IPLA* intraperitoneal local anaesthetic (intervention group), *LOE* level of evidence, *LOS* length of hospital stay, *PACU* postoperative acute care unit, *PEF* peak expiratory flow, *PONV* postoperative nausea and vomiting, *PO* postoperative, *RCT* randomised controlled trial

**Table 2:** Patients’ characteristics and surgery details

Study (Year)	BMI (kg/m^2^)		ASA class		History of pain syndromes (Yes = 1; No = 0)		Preoperative opioid use (Yes = 1; No = 0)		Cumulative opioid consumption (MED)		IPLA administration		Type of surgery		Length of surgery (min)		Revision surgery (SI = 1; No = 0)	
	**IPLA**	**CG**	**IPLA**	**CG**	**IPLA**	**CG**	**IPLA**	**CG**	**IPLA**	**CG**	**IPLA**	**CG**	**IPLA**	**CG**	**IPLA**	**CG**	**IPLA**	**CG**
Alamdari et al. (2018) [46]	44.8 ± 3.9	44.8 ± 4.2	NR	NR	0	0	0	0	NR	NR	At the end of the surgery, the peritoneal cavity, above the stomach, under the diaphragm, and the bed of the spleen were irrigated with 30 cm^3^ of 0.25% bupivacaine	At the end of the surgery, the peritoneal cavity, above the stomach, under the diaphragm, and the bed of the spleen were irrigated with 30 cm^3^ of NSS	LSG	LSG	NR	NR	0	0
Jarrar et al. (2021) [48]	48.6 ± 6.10	45.8 ± 6.07	NR	NR	1	1	NR	NR	Hydromorphone 64.902 ± 96.124 Tramadol 9.99826 ± 11.74436	Hydromorphone 57.823 ± 73.738 Tramadol 9.88844 ± 12.17774	After pneumoperitoneum and all trocars were placed, the standard suction/irrigation device was used to instil 100 mL of 0.2% ropivacaine	After pneumoperitoneum and all trocars were placed, the standard suction/irrigation device was used to instil 100 mL of NSS	LRYGB	LRYGB	1.85 ± 0.38 (h)	1.86 ± 0.40 (h)	0	0
Kaur et al. (2022) [49]	Median 42.5 (IQR 36.8–46.5)	Median 39.3 (IQR (36.7–44.9)	median 3.0 (IQR 3.0–3.0)	median 3.0 (IQR 3.0–3.0)	1	1	1 (8%)	1 (3.7%)	NR	NR	At the end of surgery, a mixing cannula was used to spray a solution of 0.2% ropivacaine (0.5 mL/kg) onto the diaphragm	At the end of each case, a mixing cannula was used to spray a solution of NSS (0.5 mL/kg) onto the diaphragm	LRYGB LSG OAGB SADI and revision surgery	LRYGB LSG OAGB SADI and revision surgery	NR	NR	1 (10%)	1 (5.6%)
Omar et al. (2019) [50]	44.18 ± 7.11	45.99 ± 8.37	NR	NR	0	0	0	0	Morphine via PCA mean ± SD (13.24 ± 7.16)	Morphine via PCA mean ± SD (16.90 ± 7.32)	Through the Veress needle or trocar, instillation in the subdiaphragmatic space of 40 mL of 0.25% bupivacaine + PSI of 0.25% bupivacaine 20 mL	Through the Veress needle or trocar, instillation in the subdiaphragmatic space of 40 mL of NSS + PSI of 0.25% bupivacaine 20 mL	LSG MGB LSG + cardioplasty (plication) LSG + cholecystectomy Diagnostic laparoscopy + adhesiolysis + plication of remnant stomach LSG + adhesiolysis Plication of remnant stomach Conversion LSG to MGB LSG + formal hiatal hernia repair		62 ± 11	61 ± 12	1 (4%)	0
Ruiz-Tovar et al. (2016) [51]	46.9 ± 9.6	44.7 ± 5.9	NR	NR	0	0	0	0	NR	NR	Instillation of 300 mg of ropivacaine in 200 mL of NSS into the abdomen after surgical dissection, just before abdominal wall closure. Under direct visualization, the solution was delivered over the oesophageal hiatus, over both anastomoses and in both subdiaphragmatic spaces. The drain was maintained clamped during the first hour after the instillation	Instillation of 200 mL of NSS into the abdomen after surgical dissection, just before abdominal wall closure. Under direct visualization, the solution was delivered over the oesophageal hiatus, over both anastomoses and in both subdiaphragmatic spaces. The drain was maintained clamped during the first hour after the instillation	LRYGB LSG	LRYGB LSG	94.8 ± 22.34	92.9 ± 23.2	1 (3.6%)	1 (3.6%)
Safari et al. (2019) [52]	43.7 ± 3.4	45 ± 4.0	II n = 54 (100%)	II *n* = 50 (96.2%); III *n* = 2 (3.8%)	0	0	0	0	Pethidine 6.88 ± 1.96	Pethidine 10.39 ± 1.35	At the end of surgery, and the absence of significant bleeding, 50 mL of 0.2% bupivacaine was poured through laparoscopic port by the surgeon to wash the operated site	At the end of surgery, and the absence of significant bleeding, 50 mL of NSS was poured through laparoscopic port by the surgeon to wash the operated site	LSG RYGB MGB	LSG RYGB MGB	NR	NR	0	0
Symons et al. (2007) [54]	48 ± 1 (SEM)	49.2 ± 1.1	NR	NR	0	0	0	0	PCA hydromorphone 27 ± 2.5	PCA hydromorphone 26.5 ± 2.5	Before incision, PSI with a 0.5% solution of bupivacaine with epinephrine. After pneumoperitoneum, 15 mL of 0.5% bupivacaine was sprayed through an instrument aimed at the oesophageal hiatus	Before incision, PSI with a 0.5% solution of bupivacaine with epinephrine. After pneumoperitoneum,15 mL of NSS was sprayed through an instrument aimed at the oesophageal hiatus	LRYGB	LRYGB	102.5 ± 5.0	104.4 ± 4.5	0	0
Zheng et al. (2023) [55]	Median 39.8 (IQR 10.79)	Median 40.8 (IQR 10.54)	NR	NR	0	0	0	0	NR	NR	10 mL of 0.7% ropivacaine instillation into the left crus dissected area	10 mL of NSS instillation into the left crus dissected area	LSG	LSG	NR	NR	0	0

*ASA* American Society of Anesthesiologists, *BMI* body mass index, *CG* control group, *IPLA* intraperitoneal local anaesthetic (group), *LRYGB* laparoscopic Roux-En-Y gastric bypass, *LSG* laparoscopic sleeve gastrectomy, *MED* morphine equivalent dose, *MGB* mini gastric bypass, *SEM* standard error of the mean, *NR* not recorded, *NSS* normal saline solution 0.9%, *OAGB* one-anastomosis gastric bypass, *PCA* patient-controlled analgesia, *PSI* port site injection, *SADI* single anastomosis duodenal-ileal bypass

### Risk of bias

**Fig. 2:**
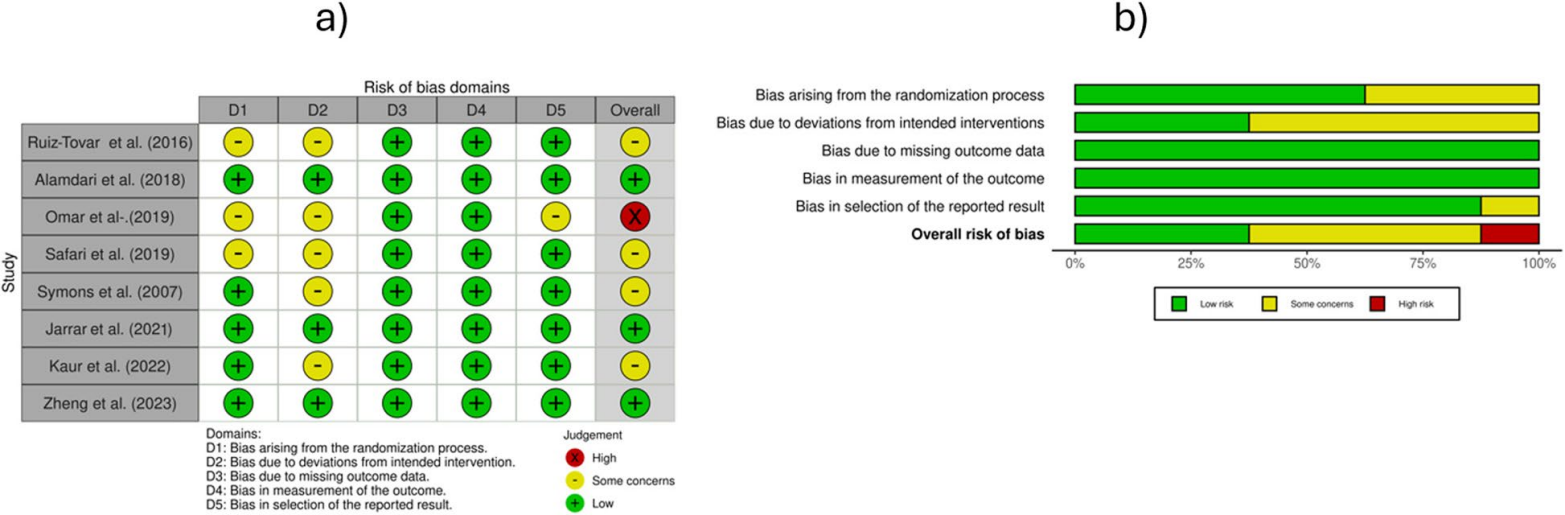
Risk of bias (RoB2). This figure presents the assessment of methodological quality across the included randomized controlled trials using the Cochrane Risk of Bias 2 (RoB2) tool. **a** The traffic light plot illustrates judgments for each domain of bias in individual studies, with green indicating low risk, yellow indicating some concerns, and red indicating high risk. **b** The summary plot shows the proportion of studies judged at different levels of risk for each domain

Three of eight studies showed a low risk of bias [46, 48, 55]. Four studies had some concerns: two had concerns related to sequence allocation concealment and unclear reporting of possible deviation from planned interventions [51, 52], while the other two lacked clear information about loss to follow-up [49, 54]. Only one study was categorized as high risk of bias [50]: although the study was declared as a randomised trial, there was neither information on the randomization procedure used nor any indication of possible deviations from the planned interventions. The complete assessment of the risk of bias is shown in Fig. 2.

### Pain

**Fig. 3:**
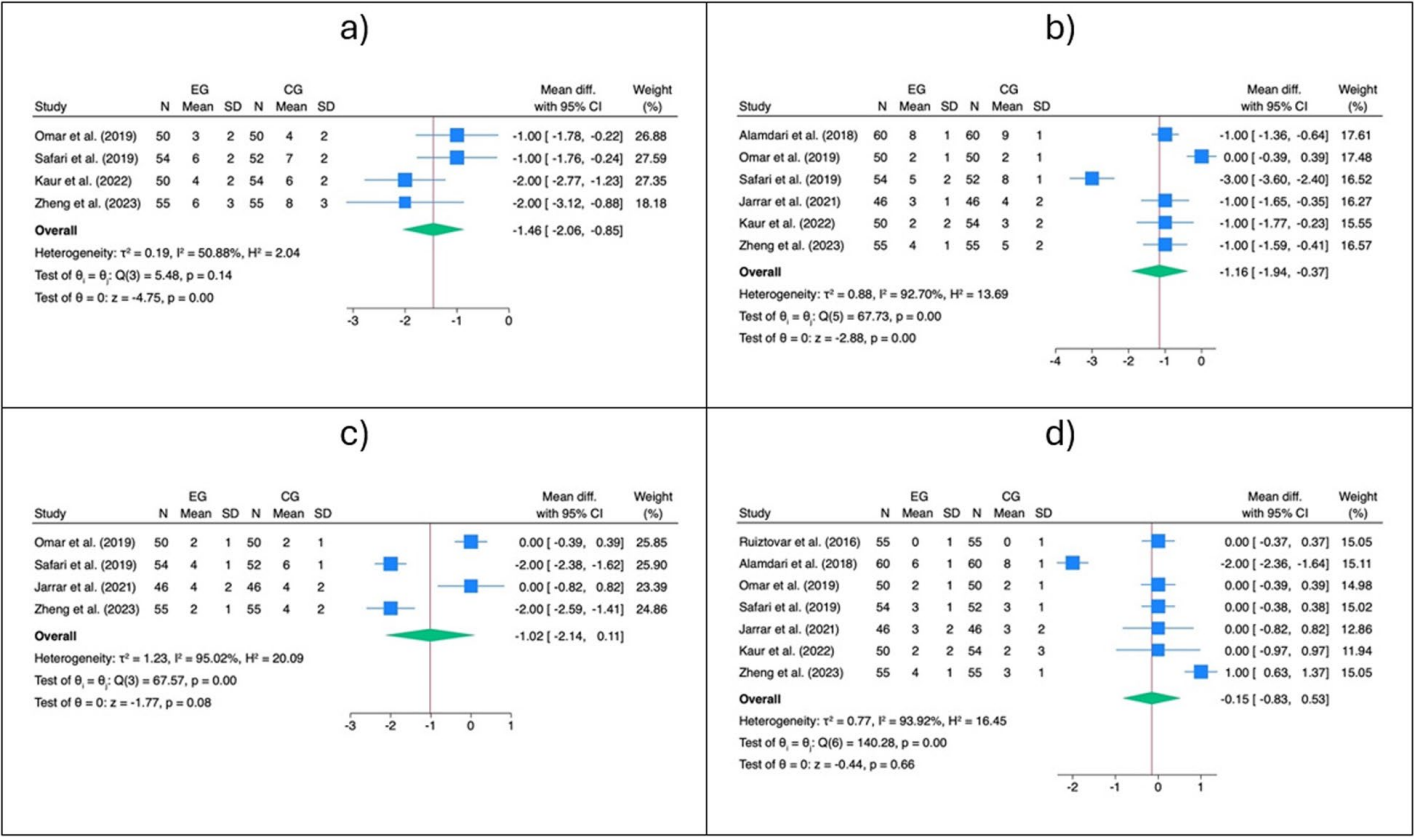
Primary outcome—pain. Forest plots showing standardized mean differences (SMDs) for postoperative pain following laparoscopic bariatric surgery comparing intraperitoneal local anaesthetic (IPLA) with control at different time intervals: **a** 0–4, **b** 4–8 h, **c** 8–12 h, and **d** > 12 h

**Fig. 4:**
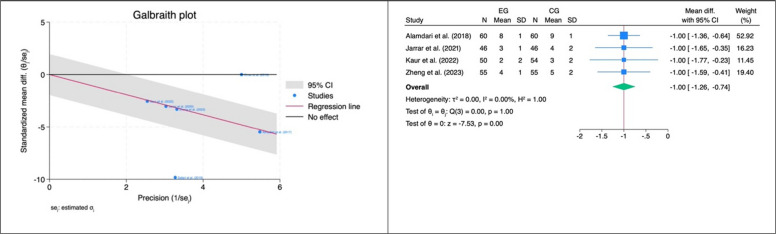
Outliers detection—outcome: pain 4–8 h. This figure identifies potential outlier studies influencing the pooled effect on postoperative pain 4–8 h after laparoscopic bariatric surgery. Each point represents an individual study’s standardized mean difference (SMD) with 95% confidence intervals

The pooled analysis of pain in the period 0–4 h after surgery showed that the IPLA group had lower pain scores compared to the CG (SMD: − 1.46, 95% CI: − 2.08, − 0.85, *z* = − 4.75, *p* < 0.001, Fig. 3a). The same trend was observed for pain measured at the 4–8 h interval (SMD: − 1.16, 95% CI: − 1.94, − 0.37, *z* = − 2.88, *p* < 0.001, Fig. 3b), although two outliers were detected in this last case (Fig. 3b). After excluding the outliers, the significant effect size in favour of the IPLA was confirmed (SMD: − 1.00, 95% CI: − 1.26, − 0.74, *z* = − 7.53, *p* < 0.001, Fig. 4). No statistically significant pooled SMD was observed for pain in the subsequent intervals (i.e. 8–12 h and more than 12 h after surgery).

**Fig. 5:**
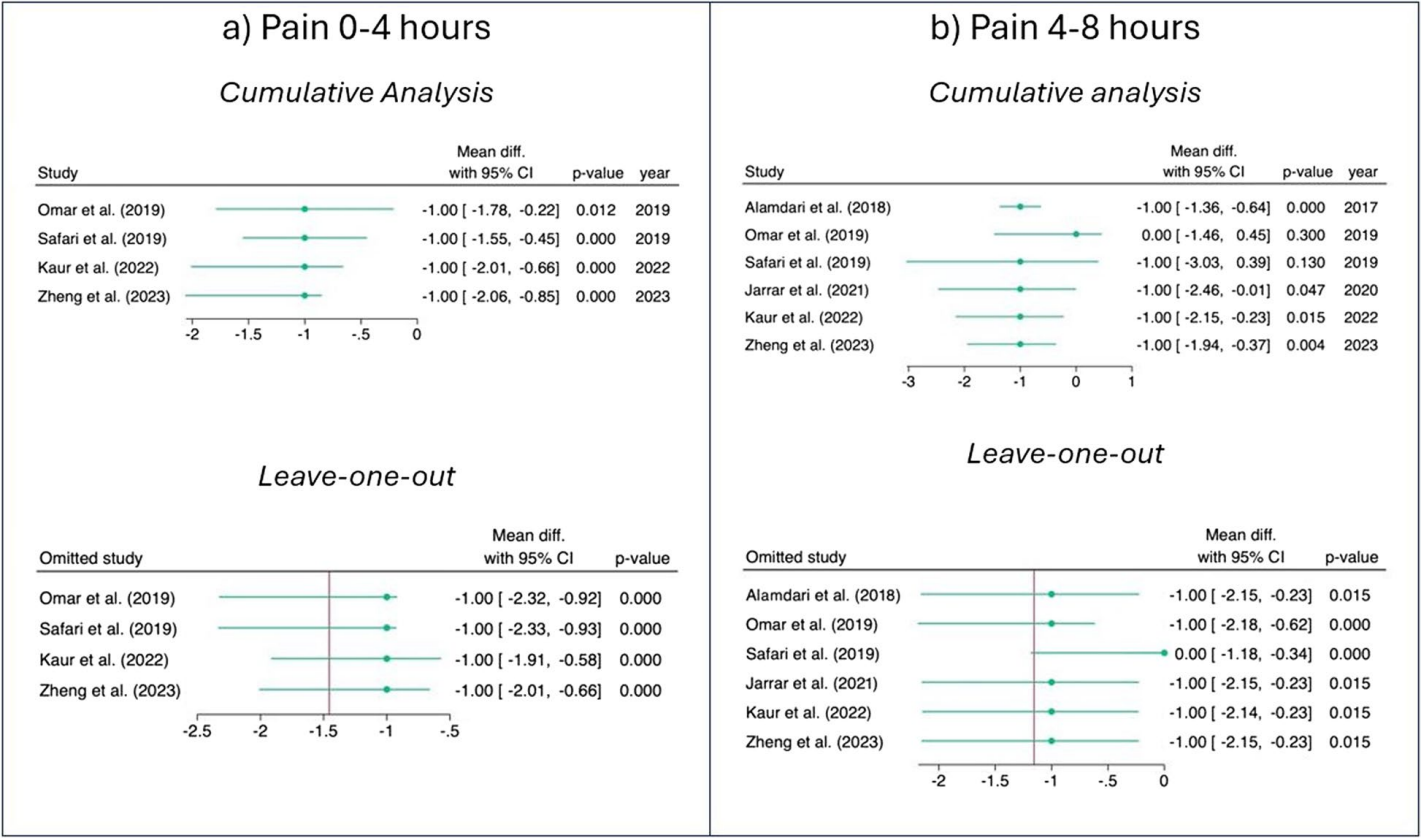
Cumulative and leave-one-out analysis. Cumulative and leave-one-out sensitivity analyses for postoperative pain following laparoscopic bariatric surgery comparing IPLA with control. Panels show: **a** Pain 0–4 h, **b** pain 4–8 h. Cumulative analysis assesses the effect of sequentially adding studies, while leave-one-out analysis evaluates the influence of each individual study on the overall pooled effect

The cumulative analysis and the leave-one-out analyses performed for pain 0–4 h did not reveal any significant changes (Fig. 5a). In contrast, the cumulative analysis for pain 4–8 h showed a stabilization of the trend over time (Fig. 5b), while the sensitivity analysis after exclusion of Safari et al. (2019) [52] showed a particular movement in the effect size.

### Secondary outcomes

**Fig. 6:**
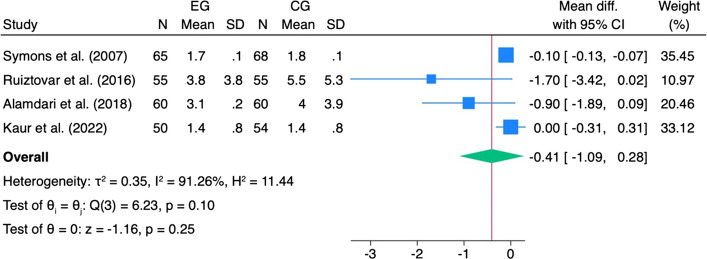
Length of stay. Forest plot showing the impact of intraperitoneal local anaesthetic (IPLA) versus control on hospital length of stay (LOS) following laparoscopic bariatric surgery. Each square represents the mean difference for an individual study, with the size proportional to study weight; horizontal lines denote 95% confidence intervals. The diamond represents the pooled effect

**Fig. 7:**
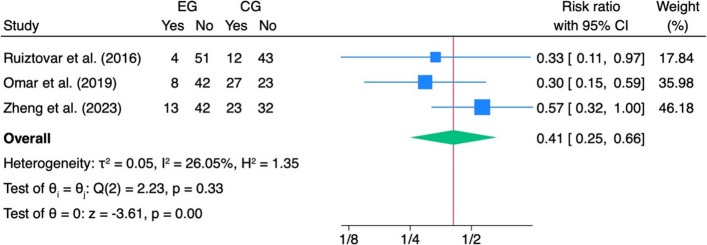
Analgesic on demand. Forest plot depicting the effect of intraperitoneal local anaesthetic (IPLA) versus control on the use of additional analgesics after laparoscopic bariatric surgery. Each square represents the risk ratio (RR) for an individual study, with the size proportional to study weight; horizontal lines indicate 95% confidence intervals. The diamond represents the pooled effect, showing that IPLA significantly reduced the need for additional analgesics

**Fig. 8:**
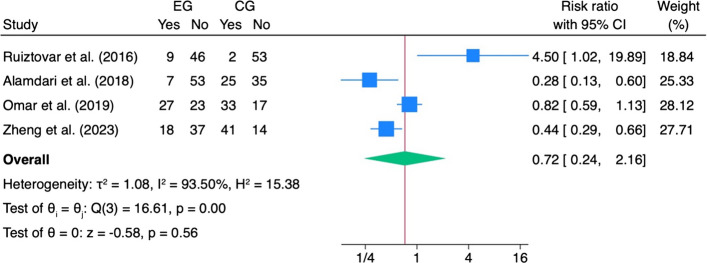
Postoperative nausea and vomiting (PONV). Forest plot showing the effect of intraperitoneal local anaesthetic (IPLA) versus control on the incidence of postoperative nausea and vomiting following laparoscopic bariatric surgery. Each square represents the risk ratio (RR) for an individual study, with the size proportional to study weight; horizontal lines denote 95% confidence intervals. The diamond represents the pooled effect, indicating no significant difference in PONV between IPLA and control groups

In patients who received IPLA, the risk ratio for the use of analgesics on demand decreased significantly (RR: 0.41, 95%CI: 0.25, − 0.66, *z* = − 3.61, *p* < 0.001, I^2^ = 26.05%) (Fig. 6). Both the length of stay and PONV showed no specific effect size in favour of the IPLA (Figs. 7 and 8).

Only four studies [48, 50, 52, 54] reported on cumulative opioid consumption. Due to the considerable variability in opioid types and administration route, meta-analysis was not possible. An analysis of the individual studies did not reveal any significant evidence of consistent differences between the two groups. Hydromorphone consumption was higher in IPLA (64.90 ± 96.12 MED) than in the CG (57.82 ± 73.73 MED) [48]. Tramadol consumption showed no statistically significant difference (IPLA: 9.99 ± 11.74 MED; CG: 9.89 ± 12.18 MED) [48]. Conversely, morphine consumption via PCA was lower in the IPLA (13.24 ± 7.16 MED) than in the CG (16.90 ± 7.32 MED) [50], as was pethidine consumption (IPLA: 6.88 ± 1.96 MED; CG: 10.39 ± 1.35 MED) [52]. PCA hydromorphone consumption was comparable between the two groups (IPLA: 27 ± 2.5 MED; CG: 26.5 ± 2.5 MED) [54].

### Certainty of evidence

The certainty of evidence was rated as moderate for the reduction in postoperative pain at 0–4 h and 4–8 h and for the reduced need for on-demand analgesia. For pain after 8 h, the certainty was downgraded to low due to inconsistency, indirectness and imprecision. The evidence for postoperative opioid consumption was also rated low, mainly due to high heterogeneity and imprecision. Outcomes such as PONV and LOS were associated with moderate certainty given the consistent results and acceptable methodological quality, although no significant differences were observed between groups. A summary of the GRADE assessment for each outcome can be found in Table 3.

**Table 3:** GRADE—level of certainty

Certainty assessment							№ of patients		Effect		Certainty	Importance
**№ of studies**	**Study design**	**Risk of bias**	**Inconsistency**	**Indirectness**	**Imprecision**	**Other considerations**	**Intraperitoneal instillation of local anaesthetic**	**Placebo or intraperitoneal anaesthetic**	**Relative (95% CI)**	**Absolute (95% CI)**		
**Pain (0–4 h)**												
8	Randomised trials	Not serious	Serious^a^	Not serious	Not serious		435	440	–	SMD **1.46 SD lower** (2.08 lower to 0.85 lower)	Moderate	Critical
**Pain (4–8 h)**												
8	Randomised trials	Not serious	Serious^b^	Not serious	Not serious		435	440	–	SMD **1 SD lower** (1.26 lower to 0.74 lower)	Moderate	Critical
**Pain (> 8 h)**												
8	Randomised trials	Not serious	Serious^c^	Serious^d^	Serious^e^		435	440	–	**0** (0 to 0)	Low	Critical
**Analgesic requirement (on-demand analgesia)**												
6	Randomised trials	Not serious	Not serious	Not serious	Not serious		25/160 (15.6%)	62/160 (38.8%)	**RR 0.41** (0.25 to 0.66)	**229 fewer per 1000** (from 291 to 132 fewer)	Moderate	Critical
**Opioid consumption**												
4	Randomised trials	Not serious	Very serious^f^	Not serious	Serious^g^				Not pooled	See comment	Low	Important
**Postoperative nausea and vomiting (PONV)**												
7	Randomised trials	Not serious	Not serious	Not serious	Serious^h^		61/220 (27.7%)	101/220 (45.9%)	**RR 0.72** (0.24 to 2.16)	**129 fewer per 1000** (from 349 fewer to 533 more)	Moderate	Important
**Length of stay (LOS)**												
5	Randomised trials	Not serious	Not serious	Not serious	Serious^e^		230	237	–	SMD **0.41 SD higher** (1.09 lower to 0.28 higher)	Moderate	Important

*CI* confidence interval, *RR* risk ratio, *SMD* standardised mean difference

^a^Substantial heterogeneity in effect sizes

^b^Outliers detected

^c^Effect inconsistent and heterogeneous

^d^Later pain less clinically relevant

^e^Wide CI, small sample sizes

^f^Different opioids, administration routes, metrics

^g^Conflicting results, small samples

^h^Wide CI crossing no effect

To improve the clinical interpretability of these results and provide context-specific guidance, 95% prediction intervals were calculated for all continuous outcomes. For postoperative pain at 0–4 h, the prediction interval ranged from − 2.08 to − 0.85 (SMD) and for pain at 4–8 h from − 1.26 to − 0.74. These intervals were almost identical to the respective confidence intervals, suggesting minimal heterogeneity and supporting the reproducibility of the early analgesic effect of IPLA in different settings. The prediction interval for length of hospital stay ranged from − 0.28 to + 1.10 (SMD), indicating substantial variability and uncertain clinical benefit. For PONV, the prediction interval (RR: 0.24 to 2.16) was wide and included both potential benefits and harms, reflecting high imprecision. No prediction intervals were estimated for on demand analgesia (binary outcome, limited number of events), although the pooled effect (RR 0.41; 95%CI: 0.25–0.66) suggests a consistent opioid-sparing effect. No pooled estimate or prediction interval could be calculated for cumulative opioid use either, due to considerable heterogeneity in reporting formats and medication types.

Overall, the GRADE assessment and prediction intervals provide a nuanced interpretation of the current evidence: the analgesic efficacy of IPLA in the immediate postoperative period appears to be robust and generalizable, while outcomes such as PONV, LOS and opioid consumption warrant cautious interpretation.

## Discussion

Pain management in bariatric surgery is a key component of postoperative care due to its impact on recovery, mobilization and global outcomes of surgery, in accordance with ERABS protocols [26, 27, 64]. Effective pain management reduces complications, minimises opioid-related side effects and facilitates early discharge [65]. IPLA has been demonstrated to be simple and effective RA technique in a variety of laparoscopic surgeries [66, 67], although its potentiality in bariatric surgery has not been sufficiently explored.

According to our findings, pain scores were significantly lower in IPLA compared to CG in the first 8 postoperative hours, while no statistically significant differences were observed after this timepoint.

Moreover, from our analysis, IPLA significantly reduces the risk ratio for the use of postoperative opioids on demand. However, due to primary data inconsistency, a meta-analysis between the two intervention groups was not feasible, although two of the included studies [50, 52] showed a reduction in total MED after surgery for IPLA. Two other studies [48, 54] showed no differences and had conflicting data about total MED after surgery between IPLA and CG.

Additionally, no specific effect size in favour of the IPLA was observed for LOS or PONV. Despite better analgesia and reduced analgesic consumption, which aligns well with ERABS recommendations, our work did not identify differences in PONV as well as postoperative clinical complications, like respiratory, cardiovascular, and infections, which were analysed only by one work ^46^.

Postoperative pain following laparoscopic surgery often results from parietal pain due to port site insertion and a visceral pain related to direct injuries and sutures and pneumoperitoneum, the latter characterized by a multifactorial origin including nerve traction from peritoneal inflation, diaphragmatic irritation from increased intra-abdominal pressure and CO_2_ insufflation. As a result, a referred shoulder pain and a vagal afferent injury associated with the formation of the “*autonomic*/*peritoneal wound*” may occur. All these factors contribute to the sickness response observed after abdominal surgery [68–70]. IPLA has shown particular efficacy in managing visceral pain by targeting the injured viscera within the peritoneal cavity rather than the abdominal wall, as demonstrated by Choi et al. [44] and Das et al. [71]. On the other hand, parietal pain can be effectively addressed with various RA techniques, such as TAP block [72], ESP block [73], QLB [35] and PSI [33, 37].

Our findings highlight the value of multimodal postoperative analgesic strategies in pain management confirming IPLA as an effective, safe and simple technique for pain management and early functional recovery after laparoscopic abdominal surgery. Consistently, the latest PROcedure-SPECific postoperative pain management (PROSPECT) guidelines recommend IPLA as the first choice for analgesia in laparoscopic cholecystectomy, further supporting its clinical relevance in minimally invasive surgery [66]. In line with PROSPECT guidelines for laparoscopic colorectal surgery [67] and laparoscopic cholecystectomy [66], we suggest to combine IPLA with PSI to optimize both somatic and visceral postoperative pain. In addition, a personalized multimodal pain management protocol may contribute to optimize postoperative analgesia and enhance quality of recovery.

### Limitations

The included studies presented several limitations. First, in three studies [51, 52], repeated measures were analysed using inappropriate statistical methods, increasing the risk of false positives by ignoring inter-individual variability and time-related changes. Future trials should apply suitable approaches, such as repeated-measures ANOVA or, preferably, linear or generalised linear mixed-effects models. Second, three studies [46, 50, 54] used parametric tests on ordinal variables like the VAS, which often violates normality assumptions. Non-parametric or ordinal regression methods are preferable to reduce bias and type I error. Third, only two studies [48, 51] reported using intention-to-treat (ITT) analysis, raising concerns about attrition bias. ITT should be the standard in future RCTs, supported by per-protocol sensitivity analyses. Fourth, clinical heterogeneity was substantial. Techniques (SG, Roux-en-Y, banding), anaesthetics (Ropivacaine vs. Bupivacaine), IPLA methods (irrigation, spraying, instillation) and timing (during or after surgery) all varied, with no clear consensus or evidence favouring one option [15]. Two protocol deviations should be noted. Our PICO included both placebo and alternative analgesic techniques, but only placebo-controlled trials were eligible. Also, planned assessment of anaesthesia-related complications was not possible due to limited data. Statistical heterogeneity was moderate to substantial for several outcomes. Meta-regression was not feasible, but qualitative analysis of factors such as procedure type, anaesthetic agent, and IPLA protocol was performed. Sensitivity and cumulative analyses supported the consistency of short-term IPLA effects. We also calculated 95% prediction intervals to estimate expected ranges in future studies; only PONV showed variability. Publication bias could not be formally assessed due to limited study numbers, though selective reporting is a concern. Trial pre-registration, data sharing and compliance with CONSORT guidelines are recommended.

Bias risk, assessed using Cochrane RoB 2.0, was low to moderate overall, though randomisation processes were often poorly described. This may have affected group comparability. Sensitivity analyses excluding higher-risk studies showed consistent results. Assessing pain at multiple timepoints is a strength. However, focusing only on the highest score per interval may limit insight into analgesic patterns. Reporting average pain scores at defined intervals (e.g. 1 h, 6 h, 24 h) and sharing patient-level data would allow better modelling. The lack of effect on PONV, despite reduced pain and opioid use, suggests the need to consider patient-specific and pharmacological factors. Future studies should stratify by comorbidities (e.g. OSAS) and baseline PONV risk. Long-term outcomes, including chronic pain, opioid use and recovery quality, should also be prioritised.

The findings of this meta-analysis support the use of IPLA to improve early postoperative pain control and reduce opioid consumption, although no significant effect was observed on PONV or complications. Evidence certainty was moderate, with considerable heterogeneity linked to surgical techniques, anaesthetic agents, and IPLA protocols. Methodological issues—including inadequate statistical methods, limited use of intention-to-treat analysis, and incomplete reporting—highlight the need for more rigorous trials. Future research should adopt advanced models, assess long-term outcomes, and include well-powered multicentre RCTs with harmonised protocols.

## Supplementary Information

Additional file 1.

## Data Availability

No datasets were generated or analysed during the current study.
