# Supplementary material for: Analgesic efficacy of intraperitoneal local anaesthetic instillation (IPLA) in laparoscopic bariatric surgery: a systematic review and meta-analysis
Source: J Anesth Analg Crit Care. 2026 Jan 26;6:33. doi: 10.1186/s44158-026-00345-3 (PMC12918028; doi:10.1186/s44158-026-00345-3)
Supplement: Supplementary file 1 — Additional file 1. [file 44158_2026_345_MOESM1_ESM.docx]

**SEARCH STRATEGY**

#1: ("intraperitoneal") OR "intra peritoneal"

#2: ((("instillation") OR "infiltration") OR "injection") OR "administration"

#3: ((((((((("local anesthetic") OR "local anaesthetic") OR "local anesthesia") OR "local anaesthesia") OR "lidocaine") OR "lignocaine") OR "mepivacaine") OR "ropivacaine") OR "bupivacaine") OR "levobupivacaine",

#4: (((("bariatric surgery") OR "sleeve gastrectomy") OR "gastric bypass") OR "roux en y") OR "gastric banding",

#5: ("laparoscopic") OR "laparoscopy"

**Scopus**

(((((("intraperitoneal") OR "intra peritoneal")) AND (((("instillation") OR "infiltration") OR "injection") OR "administration")) AND (((((((((("local anesthetic") OR "local anaesthetic") OR "local anesthesia") OR "local anaesthesia") OR "lidocaine") OR "lignocaine") OR "mepivacaine") OR "ropivacaine") OR "bupivacaine") OR "levobupivacaine")) AND ((((("bariatric surgery") OR "sleeve gastrectomy") OR "gastric bypass") OR "roux en y") OR "gastric banding")) AND (("laparoscopic") OR "laparoscopy")

**PubMed–Medline**

(((((("intraperitoneal") OR "intra peritoneal")) AND (((("instillation") OR "infiltration") OR "injection") OR "administration")) AND (((((((((("local anesthetic") OR "local anaesthetic") OR "local anesthesia") OR "local anaesthesia") OR "lidocaine") OR "lignocaine") OR "mepivacaine") OR "ropivacaine") OR "bupivacaine") OR "levobupivacaine")) AND ((((("bariatric surgery") OR "sleeve gastrectomy") OR "gastric bypass") OR "roux en y") OR "gastric banding")) AND (("laparoscopic") OR "laparoscopy")

**Web of Science**

(((((("intraperitoneal") OR "intra peritoneal")) AND (((("instillation") OR "infiltration") OR "injection") OR "administration")) AND (((((((((("local anesthetic") OR "local anaesthetic") OR "local anesthesia") OR "local anaesthesia") OR "lidocaine") OR "lignocaine") OR "mepivacaine") OR "ropivacaine") OR "bupivacaine") OR "levobupivacaine")) AND ((((("bariatric surgery") OR "sleeve gastrectomy") OR "gastric bypass") OR "roux en y") OR "gastric banding")) AND (("laparoscopic") OR "laparoscopy")

**Cochrane Library**

(((((("intraperitoneal") OR "intra peritoneal")) AND (((("instillation") OR "infiltration") OR "injection") OR "administration")) AND (((((((((("local anesthetic") OR "local anaesthetic") OR "local anesthesia") OR "local anaesthesia") OR "lidocaine") OR "lignocaine") OR "mepivacaine") OR "ropivacaine") OR "bupivacaine") OR "levobupivacaine")) AND ((((("bariatric surgery") OR "sleeve gastrectomy") OR "gastric bypass") OR "roux en y") OR "gastric banding")) AND (("laparoscopic") OR "laparoscopy")
